# Efficacy of early neonatal vitamin A supplementation in reducing mortality during infancy in Ghana, India and Tanzania: study protocol for a randomized controlled trial

**DOI:** 10.1186/1745-6215-13-22

**Published:** 2012-02-23

**Authors:** Rajiv Bahl, Nita Bhandari, Brinda Dube, Karen Edmond, Wafaie Fawzi, Olivier Fontaine, Jasmine Kaur, Betty R Kirkwood, Jose Martines, Honorati Masanja, Sarmila Mazumder, Salum Msham, Sam Newton, Maureen Oleary, Julia Ruben, Caitlin Shannon, Emily Smith, Sunita Taneja, Sachiyo Yoshida

**Affiliations:** 1 Department of Maternal Newborn Child and Adolescent Health, World Health Organization, Avenue Appia 20, 1211 Geneva 27, Switzerland; 2 Centre for Health Research and Development, Society for Applied Studies, 45, Kalu Sarai, New Delhi-110016, India; 3 Department of Infectious Disease Epidemiology, Faculty of Epidemiology and Population Health, London School of Hygiene and Tropical Medicine, London, Keppel StreetLondon WC1E 7HT, UK; 4 Department of Nutrition and Department of Global Health and Population, Harvard School of Public Health, 677 Huntington Avenue, Boston, Massachusetts 02115, USA; 5 Department of Nutrition & Public Health Intervention Research, Faculty of Epidemiology and Population Health, London School of Hygiene and Tropical Medicine, London, Keppel StreetLondon WC1E 7HT, UK; 6 Ifakara Health Institute, Mikocheni, Dar es Salaam, PO Box 78373, Tanzania; 7 Kintampo Health Research Centre, Ghana Health Service, Kintampo, PO Box 100, Brong Ahafo Region, Ghana and Department of Nutrition & Public Health Intervention Research, Faculty of Epidemiology and Population Health, London School of Hygiene and Tropical Medicine, London, Keppel Street, London WC1E 7HT, UK

**Keywords:** Vitamin A, neonatal, infant mortality, randomized controlled trial

## Abstract

**Background:**

Vitamin A supplementation of 6-59 month old children is currently recommended by the World Health Organization based on evidence that it reduces mortality. There has been considerable interest in determining the benefits of neonatal vitamin A supplementation, but the results of existing trials are conflicting. A technical consultation convened by WHO pointed to the need for larger scale studies in Asia and Africa to inform global policy on the use of neonatal vitamin A supplementation. Three trials were therefore initiated in Ghana, India and Tanzania to determine if vitamin A supplementation (50,000 IU) given to neonates once orally on the day of birth or within the next two days will reduce mortality in the period from supplementation to 6 months of age compared to placebo.

**Methods/Design:**

The trials are individually randomized, double masked, and placebo controlled. The required sample size is 40,200 in India and 32,000 each in Ghana and Tanzania. The study participants are neonates who fulfil age eligibility, whose families are likely to stay in the study area for the next 6 months, who are able to feed orally, and whose parent(s) provide informed written consent to participate in the study. Neonates randomized to the intervention group receive 50,000 IU vitamin A and the ones randomized to the control group receive placebo at the time of enrolment. Mortality and morbidity information are collected through periodic home visits by a study worker during infancy. The primary outcome of the study is mortality from supplementation to 6 months of age. The secondary outcome of the study is mortality from supplementation to 12 months of age. The three studies will be analysed independent of each other. Subgroup analysis will be carried out to determine the effect by birth weight, sex, and timing of DTP vaccine, socioeconomic groups and maternal large-dose vitamin A supplementation.

**Discussion:**

The three ongoing studies are the largest studies evaluating the efficacy of vitamin A supplementation to neonates. Policy formulation will be based on the results of efficacy of the intervention from the ongoing randomized controlled trials combined with results of previous studies.

**Trial Registration:**

**Ghana: **Australian New Zealand Clinical Trials Registry (ANZCTR) - ACTRN12610000582055**; India: **CLINICALTRIALS.GOV - NCT01138449**; Tanzania: **Australian New Zealand Clinical Trials Registry (ANZCTR) - ACTRN12610000636055.

## Background

Globally, about 9 million under five children die every year, most of them in developing countries. About two thirds of these deaths occur in the first year of life. The global community is committed to reduce child mortality by two thirds between 1990 and 2015 (MDG4). More than a third of child deaths have been attributed to maternal and child under-nutrition [1].

Vitamin A deficiency increases the risk of disease and death from severe infections in children. It is estimated that vitamin A deficiency, defined as a low serum retinol concentration (< 0.70 μmol/l), affects 190 million preschool-age children and 19.1 million pregnant women, the majority in Africa and South-East Asia [2].

Three systematic reviews and meta-analysis of RCTs indicate that vitamin A supplementation of 6-59 month old children significantly reduces all-cause mortality in this age group by about 23-30% [3-5]. Periodic high-dose vitamin A supplementation at 6-59 months of age is now recommended by the World Health Organization (WHO) for the prevention of vitamin A deficiency and for reducing the risk of death in children. However, vitamin A supplementation for 1-5 month old infants has not been shown to be beneficial in reducing infant mortality and morbidity [6-9].

There has been considerable interest in research on determining the benefits of vitamin A supplementation of infants less than 1 month (neonatal supplementation), but the results of randomized controlled trials evaluating this intervention are conflicting. A technical consultation convened by WHO in December 2008 reviewed all available evidence and recommended an additional set of randomized placebo-controlled trials to generate the evidence necessary to formulate policy for or against neonatal vitamin A supplementation. Consequently, WHO is coordinating three trials in Ghana, India and Tanzania, to examine the efficacy and safety of neonatal vitamin A supplementation. In this paper, we briefly review the available evidence regarding neonatal vitamin A supplementation, the rationale for the three ongoing trials to address the current gap in knowledge, followed by detailed presentation of the trials' design and methods for field implementation and analyses.

A small study conducted in Indonesia in 1996 found that 50,000 international units (IU) vitamin A given on the first day of life resulted in a 64% reduction in infant mortality [10]. Subsequent studies in Bangladesh and India found that 50,000 IU vitamin A given in the first two days after birth reduced mortality in the first 6 months by about 15-20% [11,12]. However, two other studies conducted in Guinea-Bissau and Zimbabwe did not find any reduction in mortality with the same intervention [13,14].

A systematic review of literature [15] identified 6 trials involving 42,508 infants. Their meta-analysis did not find a significant overall effect of neonatal vitamin A supplementation on mortality during first year of life (Pooled relative risk 0.92, 95% CI 0.75 to 1.12, P = 0.393; random effects model). Some experts suggested that the variability of effect sizes across neonatal trials is explainable by the region in which the study was conducted [16]. In the meta-regression conducted by Gogia and Sachdev, region was not a significant predictor of heterogeneity (P = 0.133) of results although the effect sizes appeared disparate (RR 1.13, 95% CI 0.90 to 1.43 in Africa and 0.82, CI 0.66 to 1.02, in Asia). They concluded that the trials were too small to make clear conclusions about regional differences and underlying biological plausibility was uncertain.

In a recently published study from Guinea Bissau, although there was no significant effect of neonatal vitamin A supplementation on infant mortality, when pooling the data with a prior study from the same setting, it appeared that boys benefited but there was a significantly harmful effect on girls' survival [17]. However, in a new meta-analysis of all published trials, including the two studies from Guinea Bissau, there was no significant difference in the effects of neonatal vitamin A supplementation on mortality among boys and girls [18].

A technical consultation convened by WHO in December 2008 reviewed all available evidence and concluded that there was insufficient evidence base currently available to make a public health recommendation for or against neonatal vitamin A supplementation. The technical consultation recommended that additional randomized placebo-controlled trials should be conducted. It was recommended that these trials should be significantly larger than the previous trials and that they should adequately represent the types of settings in which a supplementation intervention would be considered, if proven to be efficacious. In January 2009, WHO sent out a call for expressions of interest from study sites interested in conducting randomized controlled trials in newborn vitamin A supplementation. The three trials chosen in Ghana, India and Tanzania are described in this paper.

### Goal of the Ongoing Trials

The goal of these trials is to inform global policy on the use of neonatal vitamin A supplementation for reducing infant mortality in low- and middle-income countries, by answering the following research question: Does supplementation with 50,000 IU vitamin A within the first 2-3 days after birth in low- and middle-income countries reduce the risk of death in the first six months of life?

### Objectives of each trial

The primary objective of these studies is:

■ To determine if vitamin A supplementation (50,000 IU) given to neonates once orally either on the day of birth or in the next 2 days will reduce mortality in the period from supplementation to 6 months of age by at least 15% as compared to placebo.

The secondary objectives of the studies are:

■ To determine if vitamin A supplementation (50,000 IU) given to neonates once orally either on the day of birth or in the next 2 days will reduce mortality in the period from supplementation to 12 months of age.

■ To determine the efficacy of vitamin A supplementation (50,000 IU) given to neonates once orally either on the day of birth or in the next 2 days in reducing mortality in the period from supplementation to 28 days of age.

■ To determine the efficacy of the above intervention in reducing the incidence of severe morbidity defined as hospitalizations due to any illness in the period from supplementation to 6 months of age.

■ To document the potential adverse effects of vitamin A such as bulging fontanelle, vomiting, irritability, fever, diarrhoea, inability to suck or feed, convulsions or any other condition that caused parents to be concerned, in the 3 day period following administration of the supplement.

■ To determine the vitamin A status of a subsample of infants at 2 weeks and 3 months of age in the vitamin A supplementation and placebo groups.

## Methods/Design

The three trials in Ghana, India and Tanzania are independent studies with the same study design, participants, intervention, comparison and outcomes, as well as quality control and coordination mechanisms.

### Overview of Study Design

**Figure 1:**
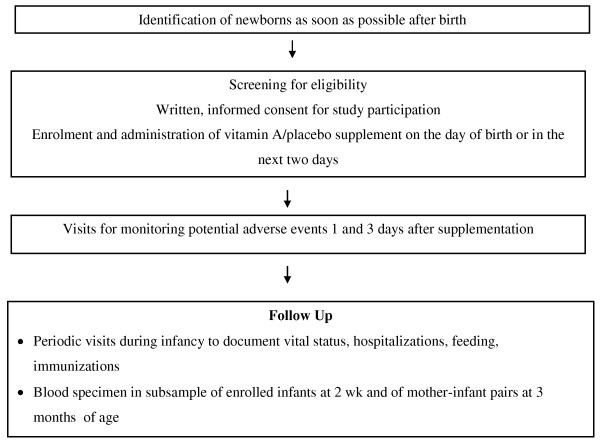
**Outline of implementation strategy common to the three studies**.

The studies are individually randomized, double-masked, placebo controlled trials. The study participants are neonates who are likely to stay in the study areas until at least 6 months of age, who are able to feed orally at the time of enrolment and whose parents provide written informed consent for study participation. Neonates randomized to the intervention group receive 50,000 IU vitamin A on the day of birth or in the next two days, and those randomized to the control group receive placebo. Potential adverse events are determined during visits by a study worker one day and three days after supplementation. A potential adverse event is defined as any harmful and/or undesired effect as reported by the family which may have resulted from the use of the vitamin A supplement. These include presence of bulging fontanelle, vomiting, fever, diarrhoea, inability to suck or feed, and convulsions. Mortality and morbidity information are collected through periodic home visits by a study worker during infancy (Figure 1).

### Sample size

The infant mortality rate was estimated to be about 50 per 1000 live births in India and about 60 per 1000 in Ghana and Tanzania. Assuming a 10% loss to follow up, sample sizes at each site were calculated to detect a 15% reduction in mortality from enrolment to 6 months of age, which is the primary outcome, with 85% power and a 5% significance level. The required sample size was 40,200 in India and 32,000 each in Ghana and Tanzania.

This sample size would also be enough to detect a difference of 15% in post-enrolment infant mortality and 20% in post-enrolment neonatal mortality (secondary outcomes).

Blood samples are requested from 720 randomly selected infants at 2 weeks of age and another 720 randomly selected infants and their mothers at 3 months of age at each site. This sample size is sufficient to detect an increase of 0.25 standard deviation (SD) in mean serum retinol in the intervention group after supplementation, with 80% power in each site, and assuming that about 30% families will refuse to allow collection of blood samples.

### Study approvals

All three study protocols were sent for scientific peer review by WHO and the suggestions of the reviewers were incorporated. The three study protocols were reviewed and approved by the WHO Ethics Review Committee. The Ghana protocol was additionally approved by the Ghana Health Service Ethical Review Committee and the ethics committees of the Kintampo Health Research Centre, and the London School of Hygiene and Tropical Medicine. The India protocol was approved by the ethics committee of the Society for Applied Studies and the state government. The Tanzania protocol was approved by the Ifakara Health Institute ethics committee, the Tanzania National Institute of Medical Research ethics committee and the Harvard School of Public Health institutional review board.

### Ethical issues

Communities in all sites were sensitized to the study prior to randomization. This was achieved through meetings with community leaders and with community groups. Informed written consent is obtained from one of the parents for each neonate before he/she is enrolled in the study. Any death within 72 hours of supplement administration, irrespective of the cause and relationship with supplementation, is communicated to WHO by the site investigators within 10 days of the team member becoming aware of the event. WHO communicates this information to the Data Safety Monitoring Board (DSMB). The DSMB reviews this information every 3 months, and all the collected data every six months to determine if the study should be continued or stopped, based on pre-decided stopping rules.

### Randomization

The unit of randomization is the individual infants. Three separate randomization schemes in blocks of size 20 are being used to allocate infants to intervention or placebo group at the three sites. The randomization lists were prepared off-site, under the responsibility of WHO. Until the close of the trial, the code will be available only to the DSMB.

### Study intervention preparation, masking and administration

A manufacturer (Strides Arcolab Limited, Bangalore, India) was identified to supply the capsules for the study in two batches. The active ingredients in the vitamin A capsules were retinol palmitate (50,000 IU) and minute amounts of vitamin E, whereas the placebo capsules contained soybean oil. The vitamin A and placebo capsules appear identical in colour, shape, and size. Capsules were individually packed in blister packs with two capsules, one for the dose and the other for the backup dose. A backup dose is provided in case of accidental spillage during administration of the main dose.

Labels were printed at WHO, Geneva, with study site and infant number in sequence (e.g. 100001 was the first number for Ghana, 200001 the first number for India and 300001 the first number for Tanzania). The labels with infant numbers allocated to the intervention group in the randomization list were fixed on capsules containing vitamin A and those allocated to the placebo group were fixed on blisters containing placebo capsules under direct supervision of WHO staff. At any time, the capsules for only one treatment arm were labelled in the packing room. When labelling and packing for the first arm was completed, the labelled blisters were placed in sealed labelled boxes and removed from the packing room before the next treatment arm capsules were brought into the room. The research teams and parents are thus completely unaware of the content of the capsules, which were only labelled with the study site and infant number.

The supplement is administered on the day of birth or in the next 2 days with a minimum period of 2 hours between birth and dosing so as not to interfere with immediate newborn care and to ensure that the infant's condition is stable. The supplement capsule is snipped with scissors near the end of the nipple and the complete contents are squeezed directly into the neonate's mouth. The backup neonate dose is administered only if the worker drops the contents accidentally after cutting the tip.

### Site description and implementation strategy of the three studies

#### Ghana

##### Study setting

The study area comprises 7 contiguous districts (Kintampo North, Kintampo South, Wenchi, Tain, Techiman, Nkoranza North and Nkoranza South) in the Brong Ahafo region of central rural Ghana, and covers 12,000 square kilometres. The study population is estimated at 600,000 and includes approximately 120,000 women of reproductive age and 15,000 live births per year. Approximately 30% of deliveries occur at home, 40% in the 4 major hospitals and the remaining 20% in a wide range of private maternity homes and small health centres across the districts. This area has a low HIV prevalence (2.6% in women attending antenatal care in 2006) and an infant mortality rate of about 60 per 1000 live births [19]. Data from previous studies in this region of Ghana indicate a Vitamin A Deficiency problem of public health significance in the area: 27.2% of 9 month old infants had serum retinol < 20 μg/dl of age [20] and 15% of pregnant women attending antenatal clinics had serum retinol < 20 μg/dl [21].

##### Identification of newborns

Village based field workers (FWs) have enumerated all houses or "compounds" in the study area and visit all reproductive age women every 3 months to identify pregnant women. Pregnant women are followed up to enable the study team to reach neonates as soon as possible after birth. The families of pregnant women are encouraged to report the birth to the study team as soon as possible, irrespective of the place of birth. Other study staff, called dosing supervisors, based in the 4 major hospitals screen all babies who are residents in the study area as soon as possible after birth for enrolment in the study. They work in close collaboration with the hospital nurses and this is an efficient method of identifying the births in the 4 major hospitals early after birth. Other dosing supervisors visit all small health centres and private maternity homes and key informants (e.g. traditional birth attendants, village based FWs) in the study area daily to identify births.

##### Screening and enrolment

The dosing supervisors assess the newborns for eligibility either in health facilities or at home. They administer the informed consent form to parents of eligible infants. They also complete a questionnaire with baseline characteristics of the mother and the family. If parents provide consent for participation of their infants in the study, the dosing supervisors allocate the next available infant number to the infant and administer the study supplement with this number. The infant is weighed and information on maternal diet, including maternal vitamin A supplementation is collected.

##### Monitoring of potential adverse events

The dosing supervisors document any potential adverse events by visits at 1 and 3 days after supplementation. While most of these visits occur at home, some are conducted in health facilities if the mother and infant are not yet discharged after a facility birth. In addition to the scheduled visits on days 1 and 3, caregivers of newborns are requested to inform the study team if they notice any unexpected clinical signs or behaviour changes in the baby within three days after supplementation. Newborns who have any adverse events are referred to the nearest health facility and managed as per standard of care of the Ministry of Health.

##### Follow up during infancy

Village-based FWs follow up enrolled infants by monthly home visits from 1 through 12 months of age. At each follow up visit, mortality events are noted and communicated to the team that conducts a verbal autopsy interview. Any hospitalizations between follow up visits are recorded. Data are collected on the infants' feeding and immunization status at each visit.

##### Blood samples

Serum samples are collected over a full calendar year (April 2011 to April 2012). Samples are collected from a subsample of infants aged 2 weeks and 3 months and the mothers of the 3 month old infants. The mothers and infants are randomly selected from the study data base using a computer generated random number list. All samples are analysed for serum retinol, retinol binding protein concentrations and C reactive protein.

##### Data management

Paper based forms are used to collect data in the field. These forms are checked by senior staff on a weekly basis and the forms are transferred to the central office every week and double entered. A SQL data management system has been developed that has in built verification, range and consistency and inter data base checks. This system also generates listings of the work load for each FW each week.

Central cleaning of the data at KHRC is performed weekly, including running additional range and consistency checks and periodic reviews of distributions and identification of outliers. Cleaned data are merged weekly with the master data set. Weekly reports are provided to the trial PIs and merged data are provided on a monthly basis to WHO.

#### India

##### Study setting

The study is being conducted in rural sites of Faridabad and Palwal districts, in the state of Haryana, India. The population of the two districts is ~2.1 million. Based on data obtained from a previous trial in the area, the birth rate in this population is 25 per thousand, the infant mortality 60 per thousand live-births per year and the less than six month mortality rate is 50 per thousand live-births per year.

The HIV prevalence among women attending antenatal care in Haryana in the year 2006 was estimated to be 0.13% [22]. Vitamin A deficiency is likely to be a problem in mothers and young children. The prevalence of subclinical vitamin A deficiency based on serum retinol < 20 μg/dl in children aged 3 to 6 years was recently reported as 14.2% [23]. Prevalence of signs and symptoms of vitamin A deficiency among children 5 to 9 years and 10 to 14 years in Haryana was reported to be 15 and 25 per thousand respectively [24]. During the study implementation period there are no programs or research on micronutrients or maternal vitamin A supplementation ongoing in the area. The national program recommends 6 monthly doses of vitamin A (100,000 IU) starting at 9 months of age; the percentage of children 12-35 months of age given vitamin A in the last 6 months in Haryana was 15.9% [25]. In the study areas, around half the families are nuclear; the median number of family members is 6 (5, 8). Around half (52%) of mothers of young children have never been to school; 95% of women do not work outside home. The median (range) family income per year in Year 2010 was Indian Rupees 36,000 (600 to 4600000). The most common sources for seeking care for ill children are private practitioners within and outside of the villages (~ 60%). Over half the deliveries are conducted at home by traditional birth attendants and over 35% of infants are born low birth weight (< 2500 g). Around 40% of under twos have < -2 HAZ and 17% have WHZ score < -2 [26].

##### Identification of newborns

The study team met community leaders, government health workers, Accredited Social Health Activists (ASHAs), Anganwadi Workers (AWWs) and Auxiliary Nurse Midwives (ANMs) and explained the purpose of the study to them. Study FWs identify pregnant women in areas allocated to them and follow them until outcome occurrence. They report births to the study coordinators. She/he allots the births to workers from the enrolment team.

##### Screening and enrolment

At enrolment, the workers explain the study to the family and in those willing, written consent is obtained from the parents of the infant. The infant is given the dose of vitamin A/placebo and forms containing baseline socioeconomic characteristics, information on feeding practices and weight of the infant and mother dietary questionnaires are filled in net books. The family is given a card containing telephone numbers of the study physicians for any further information on the study.

##### Monitoring of potential adverse events

After enrolment, each infant is visited by the enrolment team at hospital or home 1 day and 3 days after supplementation to ascertain adverse events. Newborns with adverse events are referred/escorted to the nearest health facility for management.

##### Follow up during infancy

Each enrolled infant is allocated to a home visit worker (contiguous study areas are allocated to FWs in advance) for further follow up visits till 12 months of age. All infants are contacted when aged 1, 3, 6 and 12 months by the home visit worker to document vital status, hospitalization, immunization, and information on feeding practices. Verbal autopsies are conducted to ascertain causes of deaths reported. Whenever hospital records are available these are photocopied and attached to the form. A hospitalization is defined as either an inpatient admission (where an infant receives an inpatient slip with a registration number and is allotted a bed) or a stay of ≥ 6 hours duration in the hospital including the emergency services, diarrhoea management room or any paediatric wards of the institution.

##### Blood samples

Blood specimens are being obtained from a subsample of infants at 2 weeks and mother infant pairs at 3 months for serum retinol, retinol binding protein concentrations and C- reactive proteins. Specimens will be collected over one calendar year and during one week every month. The study areas have been divided into four quadrants. Every month a blood specimen is requested from 60 randomly selected infants aged 2 weeks (up to +6 days) and 60 mother-infant pairs (infant aged 3 months up to +6 days) residing in one quadrant.

##### Data management

All data are captured electronically in net books. The data management centre is set up in the field office rented in the field sites. Range and logical checks are incorporated and cleaned data are sent to WHO every month.

#### Tanzania

##### Study setting

The study is being conducted in both urban and rural areas in Tanzania. In Dar es Salaam, the study is based in urban and semi-urban health facilities and their respective catchment areas. In Morogoro region, the study is carried out in Ifakara town and surrounding rural Kilombero, Ulanga and Kilosa districts nested within a demographic and health surveillance system. There are approximately 2.5 million people living in Dar es Salaam and 400,000 in Ifakara and surrounding areas. The 2004-05 Tanzania Demographic and Health Survey [27] estimated the infant mortality rate at 73 per 1000 live births in urban areas and 85 per 1000 live births in rural areas. The prevalence of Vitamin A deficiency for infants at age 6 months was reported to range from 26% to 41%.

##### Identification of newborns

Newborns are identified as part of a pregnancy and birth surveillance system or when an infant is born in selected labour wards in the study areas. Women recruited during pregnancy, either during routine antenatal care or through regular household visits conducted as part of the demographic and health surveillance system, are consented and followed closely during pregnancy. Information about household demographics, nutritional status, and pregnancy complications are collected during this time. For women recruited at labour wards, the same information is collected in the week following birth. For women recruited as part of pregnancy surveillance, births are identified by routine follow up by field staff, by village-based key informants, or because women self-report their own birth. All infants born in selected labour wards in the study areas are screened for enrolment.

##### Screening and enrolment

After the birth of a live infant, parents are asked for consent to participate in the study. Those who give written informed consent are screened to determine if the infant meets the eligibility criteria. Those consenting and eligible are given a single dose of 50,000 IU of vitamin A or placebo. The number written on the blister pack of vitamin A or placebo becomes the infant's ID, and an identity card is issued.

Infants are randomized individually within the geographical strata according to a computer-generated random number list, arranged in blocks of 20, in which unique identification numbers corresponds to one of the two interventions. Each new infant is assigned the next available study number. Previous experience has demonstrated feasibility to conduct the individually randomized controlled trial in these study clinics and communities.

##### Monitoring of potential adverse events

Trained FWs visit enrolled infants at home one day and three days after dosing to monitor possible side effects of vitamin A supplementation. A standard form is completed documenting symptoms of potential side effects. For each infant, reported symptoms including bulging fontanelle, diarrhoea, vomiting, fever, inability to suck or feed, and convulsions are recorded. In addition, caregivers of newborns are asked if they notice any unexpected clinical signs or behaviour changes in the baby since the time of supplementation. If a problem is reported, study staff refers the family to the nearest health facility, where any morbidity is managed per the standard of care established by the Ministry of Health in Tanzania.

##### Follow up during infancy

Field staff visit the home of enrolled infants at 1 month, 3 months, 6 months, and 12 months after birth. The main purpose of the visits is to ascertain survival status during infancy. Additionally, information about symptoms of illness in the past month (cough, refusal to eat, fever, difficulty breathing, chest retraction, convulsions, vomiting, and diarrhoea) and any past morbidity that resulted in hospitalization are collected. For hospitalizations, detailed information is sought from medical records or mother's report about the date of admission, length of hospital stay, and the reason for admission.

During the visits, FWs assess if the child is alive and still a resident in the study area. Key informants within the demographic surveillance areas also ascertain vital status and report it to the study team. In case of a death of a study child, the event is documented, and trained field staff completes a verbal autopsy with the child's primary caregiver. For women who travel out of study area, attempts to maintain contact with her through cell phone or relatives in the area are made in order to collect information on the child's vital status.

##### Blood sample

A blood sample is requested from 728 infants at 2 weeks of age, and samples are also requested from 728 mother-infant pairs at 3 months postpartum. The samples are collected over the course of one year to cover all seasons, and specific geographical zones are sampled on a rotating basis in order to facilitate logistics. Thus, every week 14 infants that meet the age requirements and live within the designated geographical area are randomly selected. Samples are centrifuged, and serum aliquots are frozen until testing of samples.

##### Data management

Data are captured electronically using touch screen tablet PCs and net books. Software for real-time data entry has detailed range and consistency checks to ensure data quality. Data are uploaded to the main server in Dar es Salaam via the cell network or wireless internet where possible. Data are further reviewed by data management team, and identified discrepancies are resolved by site supervisors or the field coordinator. Data are uploaded each month to WHO.

### Standardization and quality assurance of the three studies

#### Data collection instruments

Across the three studies, there are shared questionnaires that each contains a set of "core" variables collected by interview, examination, anthropometry or laboratory analyses. Each core variable has a standard definition, acceptable range limits, and is maintained in agreed upon data formats.

#### Training and standardization

Research staff of all three studies has been carefully trained to conduct surveillance, interview families, obtain informed consent, administer the intervention/placebo capsules, collect baseline and follow up information and in electronic data capture (India and Tanzania).

#### Supervision and quality checks

To ensure quality of data, spot checks are done for each FW at least once a month to confirm whether the workers are visiting families. Workers who have conducted a home visit in the last 6 days are selected and families are called or visited to confirm whether the worker had visited them.

Additionally supervised visits are conducted for each worker once a month to assess and improve the quality of activities done by the worker. Different activities (consenting, screening, capsule administration, post dosing and follow up visits) conducted by workers are observed by supervisors by accompanying FWs on these visits. Each worker is accompanied at least once a month. During supervised visits the following aspects are evaluated; worker appearance and quality of interaction with the family, whether the questions in the form are asked correctly and procedures, if relevant (e.g. weighing and capsule administration), are performed correctly.

#### Site monitoring

WHO technical staff conducts at least two monitoring visits to each site every year. A detailed structured review of study implementation is done at each visit. The monitors make direct observations of study implementation and data management activities. The key areas that are monitored include the pace of recruitment, consent procedures, storage and use of study supplements, follow up visits, data collection, collection transport and storage of blood samples and data management. The recommendations arising from the site visit are discussed for implementation with the Principal Investigators.

#### Data monitoring

Data range and consistency checks are built into the data entry system and any discrepancies are returned to the field for verification. Data from all studies are sent every month to a central data repository which is established at WHO. Data quality checks are applied on a monthly basis and feedback provided to the study sites.

#### Capsule testing

The vitamin A capsule supplier provided evidence of rigorous stability testing and the certificates of analysis. Additionally an independent laboratory checks to confirm the content of the capsules throughout the study. Every three months, twenty randomly selected unused backup capsules are selected from among those capsule strips used for enrolments in the previous three months and sent to an independent laboratory for analysis of vitamin A content (VITAS, Norway).

#### Laboratory standardization

Blood samples are collected, transported and analysed according to a quality control/assurance protocol to assure retinol contents within accepted ranges. Laboratories performing analysis of blood samples for vitamin A concentrations participated in the VITAL-EQA program from the US Centers for Disease Control and Prevention (CDC) for quality control.

### Analysis plan for the three studies

#### Definitions

The primary outcome of the study is defined as a death between supplementation and 180 days of life (6 months). Secondary mortality outcomes are defined as a death between supplementation and 28 days of life, and a death between supplementation and 365 days of life. Severe morbidity is defined as one or more hospitalizations due to any illness between supplementation and day 180 of life. A hospitalization is defined as an admission to hospital as an inpatient, as reported by the mother. Infants will only contribute one hospitalization episode to the analysis, regardless of how many times they were hospitalized in the first six months of life.

Potential adverse events are defined as specific events occurring within 3 days of supplementation. These events include all deaths within 3 days of supplementation; bulging fontanelle confirmed by the research team through a physical examination of the infant; or primary caregiver reports of fever, vomiting, diarrhoea, inability to suck or feed, or convulsions in the first 3 days post supplementation.

#### General principles for analysis

All analyses will be conducted on an intention-to-treat basis, regardless of whether the infant is known to have received a full dose or not (for example, if the infants spit up all or some of the dose). All analyses will be performed with both person time (infant years of follow up) (primary analysis) and live births as the denominator. Random effects models and robust standard errors will be used to account for clustering of deaths with multiple births.

Separate analysis workshops will take place at the end of follow up in each of the three study sites. Data cleaning for the analysis of all the primary and secondary outcomes will be completed before the workshops and the databases will be locked. The randomization code will be provided to the members of the trial teams during the workshop. The code will first be provided as dummy variables (e.g. × and Y). After all analyses are completed the code will be provided as allocation groups (vitamin A and placebo). Analysis will be performed separately by site.

Individual site results will be presented to the DSMB at a joint meeting within 3 months of analysis in each site.

#### Flow of participants

**Figure 2:**
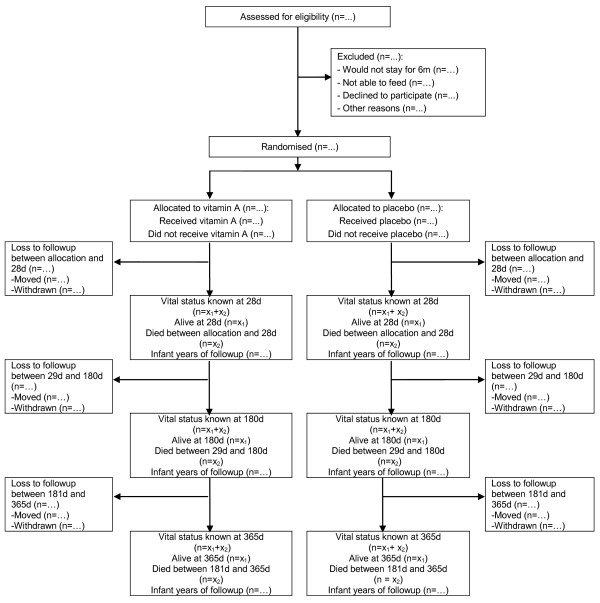
**Structure of the study flow chart**.

The flow of the number of participants through assessment of eligibility, randomization, follow-up and analysis will be documented (Figure 2). Reasons for exclusions and withdrawals will be described.

#### Comparability of participants in intervention and control groups

Summary values (means, proportions) for several background characteristics in the intervention and control groups will include maternal age, parity, maternal education, wealth index of the household, place of delivery, singleton or multiple birth, sex of infant, birth weight, median age at supplementation and immunization status (time of first DTP immunization). No significance tests will be performed [28].

#### Main effects

For the primary outcome, all-cause mortality between supplementation and 180 days of age, data will first be analysed using person time, i.e. infant years of follow up, as the denominator. Hazard ratios and 95% confidence intervals comparing the effect of vitamin A on deaths in the first half of infancy between vitamin A and placebo groups will be calculated using Cox regression. In the unlikely event that there is imbalance between the treatment and control groups, the final statistical models will include adjustment for all imbalanced variables. The effect of vitamin A supplementation on all-cause mortality between supplementation and 180 days of age will also be assessed using enrolled infants as the denominator. The principles outlined above will be followed, and logistic regression will be used to compare mortality in the vitamin A and placebo arms.

The effect of vitamin A supplementation on secondary outcomes (all-cause mortality from supplementation to 12 months, all-cause mortality from supplementation to end of the neonatal period, hospitalizations) will be assessed using similar methods to the primary analysis; both person time and enrolled infants will be used as denominators for all analyses.

#### Adverse events

Adverse events in the vitamin A and placebo groups will be tabulated [29]. The denominator will be all enrolled infants. The absolute risk of an adverse event per arm and per adverse event type will be presented.

#### Sub-group analyses

The sub-group analyses listed below were specified *a priori *in the trial protocol.

The effect of vitamin A supplementation on all-cause infant mortality from 6 to 12 months by:

- Birth weight (< 1.5 kg, 1.5-1.9 kg, 2.0-2.4 kg, ≥ 2.5 kg, and as a continuous variable)

- Sex (male or female)

- Risk of death before and after receipt of first dose of DTP vaccine. For this analysis, person-time for each infant will be divided into before and after receiving DTP vaccine.

- Economic group (richest to poorest wealth quintiles, using principal components analysis)

- Maternal large-dose vitamin A supplementation (received or not received)

Site-specific results will be presented for all subgroups. Statistical interactions will be assessed using likelihood ratios tests for unadjusted hazard ratios or Wald tests for adjusted hazard ratios. If significance tests yield a p value < 0.01 then site specific mortality rates and hazard ratios comparing mortality in the vitamin A supplementation and placebo group in each sub-group will also be reported.

#### Serum retinol analyses

The effect of vitamin A supplementation on vitamin A status will be assessed on a sub-sample of infants at 2 weeks (to establish whether there has been a short term improvement in vitamin A status) and 3 months (to establish whether there has been a longer term improvement in vitamin A status). Mean serum vitamin A and retinol binding protein concentrations at each site will be compared in the vitamin A and placebo groups using independent sample t-tests for the overall sample. If the distributions are found to be skewed, the mean log concentrations will be compared. Further analyses will be conducted in which retinol levels will be adjusted for CRP, which is a biochemical marker of infection. Maternal serum retinol concentrations will be analysed to assess the prevalence of maternal vitamin A deficiency.

#### Interim analyses by the DSMB

A common DSMB has been set up to monitor the progress of the three trials. The DSMB is responsible for monitoring and assessing the safety of the trial and consists of an epidemiologist, a statistician and a clinician/social scientist from each of the three study sites. It meets every six months, and all three trials are discussed at the same meeting. The DSMB examines adverse events and deaths within 3 days of supplementation every 3 months. In addition, there will be two formal interim analyses of mortality to six months for each site. The first interim analysis will take place when approximately one third of participants have reached six months of age and the second interim analysis will take place when approximately half of the participants have reached that point.

The analysis by DSMB is conducted in a blinded manner (treatment groups X and Y). The randomization code will be broken if there is clear evidence of a difference in mortality between the two arms. A trial will be stopped if there is early evidence of a significantly lower mortality in the vitamin A group compared with placebo according to P values specified in the O'Brien- Fleming rules [30]. For the first interim analysis, a P value of < 0.001 is specified; for the second, the specified P value is < 0.015. Only the trial centre showing increased or decreased mortality with these specified p values will be stopped; the other trial centre/s will continue until the next interim analysis or the final analysis as appropriate. For the final analysis for each site, an increase or decrease in mortality in either group will be considered significant if the P value is < 0.047; this lower value is specified to account for the fact that the data will have been examined twice before the final analysis [30].

### Timelines

Enrolment of participants started between June and August 2010 in the three studies and is likely to be completed by September 2012. After enrolment is completed, follow up visits will continue to be conducted for a period of 6 months. This will ensure that all enrolled infants have a chance to complete their 6 month follow up. A proportion of enrolled infants will therefore have less than 12 months follow up.

## Discussion

The three ongoing studies described in this paper are the largest studies evaluating neonatal vitamin A and are expected to provide definitive information to help in formulating policy in favour of or against this intervention. All studies are individually randomized, double blind placebo controlled trials. Each is individually powered to detect a moderate but important effect on mortality in enrolled infants.

The studies are being conducted in Ghana, Tanzania and India and combined with previous studies (conducted in Indonesia, Bangladesh, Nepal, India, Guinea-Bissau and Zimbabwe), will provide information generalizable to Africa and South Asia. There are very few exclusion criteria because the studies are expected to answer a pragmatic question about the effect of routine use of neonatal vitamin A.

The intervention is a single dose of 50,000 international units within the first three days of life, consistent with the previous studies that have tested this intervention. The comparison is placebo with blinding achieved through numbered supplements. This is the most robust design available to ascertain efficacy of an intervention.

The primary outcome for this study was chosen to be mortality in the period from supplementation to 6 months of age. This was done because there is a policy on vitamin A supplementation in the second half of infancy in many developing countries, and the effects of the intervention (neonatal vitamin A supplementation) will be changed by this large supplement provided in the second half of infancy. However, two previous studies from Guinea-Bissau have suggested that the intervention may have a negative effect in the second half of infancy. Therefore, we have added mortality from supplementation until 12 months of age as a secondary outcome in consultation with the DSMB of the study.

We have several subgroup analysis decided *a priori*. These subgroup analyses are planned to confirm or refute hypotheses raised by previous studies and systematic reviews. A pooled analysis of two studies from Guinea-Bissau raised the hypothesis that the intervention may have a negative effect in girls, but a meta-analysis of all available trials did not find this interaction. The studies from Guinea-Bissau have also raised the hypothesis that effects may be different before or after the first dose of DTP immunization. A meta-analysis hypothesized that the effects may be different in low or normal birth weight infants and by the level of vitamin A deficiency in populations.

The biological rationale for the potential effect of neonatal vitamin A is not known. It has been hypothesized that the intervention could work through positively affecting the immune system or by promoting the maturation and integrity of the epithelium. A set of parallel animal and human studies is being conducted to understand the transport and metabolism of the neonatal vitamin A dose and its effects on the innate and adaptive immune system. These studies will shed more light on the potential biological mechanisms through which this intervention may work.

However, policy formulation will largely be based on the results of efficacy of the intervention from the ongoing randomized controlled trials. These results will be combined with results of previous studies in a new meta-analysis for an overall effect. The data from the three new studies, as well as previous studies if possible, will be pooled to carefully examine subgroup effects.

### Trial status

The trials are ongoing. Participant recruitment is expected to be completed by September 2012.

## Abbreviations

DSMB: Data Safety Monitoring Board; DTP: Diphtheria Tetanus Pertussis; FW: Field Worker; HAZ: Height-for age Z score; IU: International Units; KHRC: Kintampo Health Research Centre, Kintampo, Ghana; MDG4: Millennium Development Goals 4; RCT: Randomized Controlled Trial; SQL: Structured Query Language; VITAL-EQA program: External quality assurance for nutritional markers; WHZ: Weight for age Z score; WHO: World Health Organization.

## Competing interests

None of the authors have any conflicts of interest. RB, JM, OF and SY are staff members of the World Health Organization. The opinions expressed by him/her in this paper are his/her own and are do not necessarily reflect the policy of the Organization

## Authors' contributions

The study proposal was developed by all the authors of the paper. Site authors along with members of the study group from each site are implementing the study. WHO staff is providing technical support, monitoring and coordination for the trials. The manuscript was prepared jointly by all authors during a workshop and has the final approval of all authors.

## Authors' information

### Ghana Authors

KE, MBBS, MSc, PhD, FRCPCH, Senior Lecturer, London School of Hygiene and Tropical Medicine, United Kingdom

SN, MBBS, MSc, PhD, Lecturer, Kintampo Health Research Centre, Ghana and London School of Hygiene and Tropical Medicine, United Kingdom

CS, MPH, Lecturer, London School of Hygiene and Tropical Medicine, United Kingdom

MO, BSc MSc, Research fellow/Trial Epidemiologist, London School of Hygiene and Tropical Medicine, United Kingdom

BK, MSc, FFPH, FMedSci, Professor of Epidemiology & International Health

London School of Hygiene and Tropical Medicine, United Kingdom

### India Authors

SMa, MD, PhD, Research Coordinator, Centre for Health Research and Development, Society for Applied Studies, New Delhi, India

ST, MD, PhD, Research Coordinator, Centre for Health Research and Development, Society for Applied Studies, New Delhi, India

JK, MSc, Coordinator, Centre for Health Research and Development, Society for Applied Studies, New Delhi, India

BD, MSc, Coordinator, Centre for Health Research and Development, Society for Applied Studies, New Delhi, India

NB, MD, PhD, Director, Centre for Health Research and Development, Society for Applied Studies, New Delhi, India

### Tanzania Authors

HM, BSc, MSc, PhD, Director of Research Programs Ifakara Health Institute, Tanzania

ES, MPH, International Research Coordinator, Harvard School of Public Health, USA.

JR, MPH, Senior Research Coordinator, Harvard School of Public Health, USA.

SMs, BSc, MPH, Global Infectious Disease research training fellow, Harvard School of Public Health, USA.

WF, MBBS, MPH, MS, DrPH, Professor of Nutrition, Epidemiology and Global Health, Harvard School of Public Health, USA.

### Coordination

RB, MBBS, MD (Pediatrics), Ph.D, Medical officer, Department of Maternal, Newborn, Child and Adolescent Health, World Health Organization, Switzerland.

JM, Ph.D MSc, MD, Coordinator, Department of Maternal, Newborn, Child and Adolescent Health, World Health Organization, Switzerland.

OF, MD, Medical officer, Department of Maternal, Newborn, Child and Adolescent Health, World Health Organization, Switzerland.

SY, MPH, Technical officer, Department of Maternal, Newborn, Child and Adolescent Health, World Health Organization, Switzerland.
